# Optical Temperature Sensing With Infrared Excited Upconversion Nanoparticles

**DOI:** 10.3389/fchem.2018.00416

**Published:** 2018-09-24

**Authors:** Kory Green, Kai Huang, Hai Pan, Gang Han, Shuang Fang Lim

**Affiliations:** ^1^Department of Physics, North Carolina State University, Raleigh, NC, United States; ^2^Department of Biochemistry and Molecular Pharmacology, University of Massachusetts Medical School, Worcester, MA, United States

**Keywords:** upconversion thermal sensing, pulsed excitation, 800 nm, 980 nm, local thermal heating, DNA denaturation

## Abstract

Upconversion Nanoparticles (UCNPs) enable direct measurement of the local temperature with high temporal and thermal resolution and sensitivity. Current studies focusing on small animals and cellular systems, based on continuous wave (CW) infrared excitation sources, typically lead to localized thermal heating. However, the effects of upconversion bioimaging at the molecular scale, where higher infrared intensities under a tightly focused excitation beam, coupled with pulsed excitation to provide higher peak powers, is not well understood. We report on the feasibility of 800 and 980 nm excited UCNPs in thermal sensing under pulsed excitation. The UCNPs report temperature ratiometrically with sensitivities in the 1 × 10^−4^ K^−1^ range under both excitation wavelengths. Our optical measurements show a ln(I_525_/I_545_) vs. 1/T dependence for both 800 nm and 980 nm excitations. Despite widespread evidence promoting the benefits of 800 nm over 980 nm CW excitation in avoiding thermal heating in biological imaging, in contrary, we find that given the pulsed laser intensities appropriate for single particle imaging, at both 800 and 980 nm, that there is no significant local heating in air and in water. Finally, in order to confirm the applicability of infrared imaging at excitation intensities compatible with single nanoparticle tracking, DNA tightropes were exposed to pulsed infrared excitations at 800 and 980 nm. Our results show no appreciable change in the viability of DNA over time when exposed to either wavelengths. Our studies provide evidence for the feasibility of exploring protein-DNA interactions at the single molecule scale, using UCNPs as a reporter.

## Introduction

**Figure 1:**
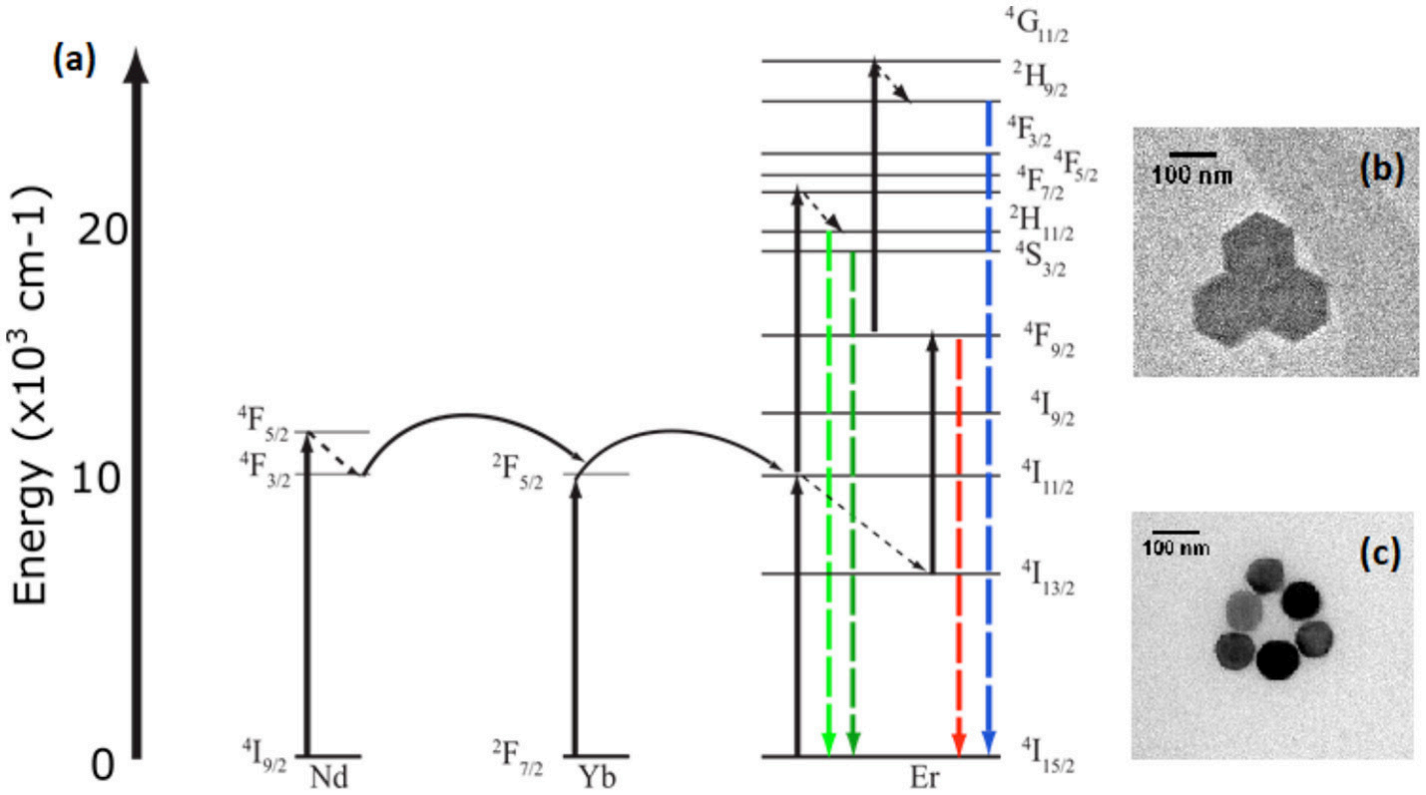
Energy level diagram **(a)** of the sensitization of Erbium by Neodymium and Ytterbium at 806 nm and 976 nm excitation respectively. Insets of transmission electron microscopy of both **(b)** β-NaYF_4_:20%Yb, 2% Er and **(c)** β-NaYF_4_:40%Yb, 2%Er@NaYF_4_:20%Yb@NaNdF_4_:10%Yb particles.

Many biological processes occurring within intracellular structures may result in changes in the pH, temperature and electrical potential, to name a few. Exploring thermal changes at the cellular level provides insight into biochemical reactions taking place in a cell. Fluorescent thermometry relies on changes of relative fluorescent intensities, lifetimes and wavelengths to local temperature (Engeser et al., 1999; Sakakibara and Adrian, 1999; Ross et al., 2001; Wang et al., 2002; Löw et al., 2008; Binnemans, 2009; Vetrone et al., 2010a). Conventional fluorescence microscopy uses short-wavelength (UV-blue) excitation, and detection of a longer-wavelength, Stokes-shifted fluorescence (Stokes, 1852; Lichtman and Conchello, 2005). However, this use of short-wavelength excitation leads to autofluorescence, photobleaching, and photodamage to biological specimens (Giloh and Sedat, 1982). Molecular dyes bleach under intense illumination (Shaner et al., 2008). Semiconductor nanoparticles (i.e., Qdots) (Tessmer et al., 2013) are stable, but display blinking and toxic behavior (Yao et al., 2005). In contrast, our method is based on rare-earth ion doped, upconversion nanoparticles (UCNPs) (Lim et al., 2006, 2009, 2010; Austin and Lim, 2008; Ungun et al., 2009). UCNPs absorb at 800 and 980 nm in the near infrared (NIR), exhibit no bleaching, are non-toxic, and are not affected by blinking (Chen et al., 2006; Schubert et al., 2006). Due to this lack of bleaching, UCNPs are well suited for long term monitoring of biological events at the high laser intensity levels employed in single cell imaging, as opposed to a dye that may bleach over time. The demonstration of UCNPs as nanothermometers in water has been shown by Vetrone et al. (2010a) and others (Chen et al., 2016; Zhu et al., 2016). The electrons in the 4f shell of rare earths are shielded from the surroundings by the filled 5s and 5p shells, and therefore the influence of the surrounding matrix on the optical transitions within the 4f shell is small, whether in crystals or in solution. Therefore, UCNPs show reduced sensitivity to physiological changes such as salt concentration (Gota et al., 2009) and pH while monitoring cellular temperatures (Vetrone et al., 2010a,b). UCNPs have also been used to measure the temperature of the interior nanoenvironment of magnetically heated iron oxide nanoparticles (Dong and Zink, 2014) and have been shown to enable direct measurement of the local temperature with high temporal (millisecond) and thermal resolution (0.3–2.0 K) (Debasu et al., 2013) and (10^−5^ K^−1^) sensitivity (Xu et al., 2012) with simple equipment requirements. The emission of the dopant ions is sensitive to temperature in some configurations due to closely spaced energy levels being thermally coupled (Bai et al., 2007; Lü et al., 2011; Xu et al., 2012; Debasu et al., 2013). Moreover, these thermally coupled energy levels are not sensitive to other environmental factors such as scattering or tissue autofluorescence. Thermally coupled emissions, such as for the Er^3+^ rare earth ion, can be in the visible, such as in the intensity ratio (RHS) of the ^2^H_11/2_ to ^4^I_15/2_ (525 nm) over ^4^S_3/2_ to ^4^I_15/2_ (545 nm) transitions (Bai et al., 2007; Lü et al., 2011; Figure 1), or in the red to near infrared, with the Tm^3+^ rare earth ion, such as in the intensity ratio (RHS) of the ^3^F_2, 3_ to ^3^H_6_ (700 nm) over the ^3^H_4_ to ^3^H_6_ (800 nm) transitions (Xu et al., 2012). In the aforementioned emissions, the energy separation between the nearest excited states Er: ^2^H_11/2_ and Er: ^4^S_3/2_, is only several hundred wavenumbers. Thus, the population distribution of Er: ^2^H_11/2_ and Er: ^4^S_3/2_ is influenced by both thermal distribution and nonradiative relaxation. As a consequence, the population of the Er: ^2^H_11/2_ level varies as a function of the Boltzmann's distribution between the two states (Lei et al., 2005). Similarly, the small energy separation between the thermally coupled Tm^3+^ levels of about 1,850 cm^−1^, gives rise to the same phenomenon (Xu et al., 2012). Measurements of the Boltzmann distribution between the two closely spaced states with varying temperatures show that the natural log of this ratio is inversely proportional to the temperature in the range relevant to most biological systems (Vetrone et al., 2010a).

However, primary excitation of the UCNPs occurs in the near infrared, where the absorption coefficient of the water abundant in biological tissue varies, resulting in some reservations regarding the use of these nanoparticles as a temperature sensor. Those reservations arise from the fact that the absorption coefficient of water at 980 nm is about 20 times larger than that at 800 nm (Weber, 1971; Wang et al., 2013). Specifically, at 980 nm CW excitation, thermal heating of the biological environment, may hamper the measurement process, as seen in small animals, and in cellular systems (Vetrone et al., 2010a; Wang et al., 2013). The more widely used sensitizer, the Yb^3+^ rare earth ion absorbs primarily at 980 nm, corresponding to the ^2^F_7/2_ to ^2^F_5/2_ transition, whereas the Nd^3+^ rare earth ion sensitizer absorbs at 800 nm, corresponding to the ^4^I_9/2_ to ^4^F_5/2_ transition. The Nd^3+^ ion has an absorption cross section an order of magnitude greater at 800 nm than the Yb^3+^ sensitizer(Kushida et al., 1968; Wang et al., 2013; Chen et al., 2015). Most studies focus on small animals and cellular systems, where the infrared excitation source is a continuous wave (CW) laser operating at low intensities. This has led to the prevailing belief that the benefits of bioimaging with CW infrared excitation at 980 nm, with low scattering background, is offset by the thermal cost of cellular heating. At this scale, 800 nm CW excitation has been shown to alleviate some of the issues with heating(Wang et al., 2013). However, given the emergent application of infrared imaging at the single molecule level, efforts to examine the thermal effects of pulsed excitation at 800 and 980 nm have never been made. Infrared upconversion bioimaging at the molecular scale, occurs at a much higher intensity under a tightly focused excitation beam, and is normally coupled with pulsed excitation to provide higher peak powers, for sharper discrimination along the Z axis. Therefore, in this work, particular emphasis is paid to the potential thermal effects of upconversion bioimaging at the molecular scale, under pulsed infrared excitation. An example of single upconversion nanoparticle imaging is shown in Supplementary Video 1. Furthermore, we address the proposed potential benefits of pulsed excitation infrared imaging at the single molecule scale, first through spectroscopic studies, and then in single molecule viability studies using DNA tightrope, under pulsed laser excitation at high magnification and intensities. A long-term single molecule imaging technology that is resistant to photobleaching and is excited at longer wavelengths can be a powerful tool to study physiological processes that take time to unfold, such as disease progression.

To that end, comparisons are made between excitations at 980 nm (β-NaYF_4_:20%Yb, 2%Er UCNPs) and 800 nm (β-NaYF_4_:40%Yb, 2%Er@NaYF_4_:20%Yb@NaNdF_4_:10%Yb core-shell-shell UCNPs), where the thermal response of the UCNP is recorded as a thermally responsive intensity ratio variation in the spectra, with no fluorescence intensity quenching, and with simple equipment requirements. In contrast to other researchers, where both I_525_ and I_545_ emission lines are derived from a spectral scan over the appropriate wavelength range, we collect both lines simultaneously, in order to avoid heat dissipation effects. Our optical measurements show a ln(I_525_/I_545_) vs. 1/T dependence for both 800 nm and 980 nm excitations, in the Yb^3+^/Er^3+^ codoped and triply doped Yb^3+^/Er^3+^@Yb^3+^/Nd^3+^ samples. We also observe a strong influence of the laser intensity on the relative spectroscopic ratio of I_525_/I_545_. Additionally, we find that given the pulsed laser intensities appropriate for single particle imaging, at both 800 and 980 nm, that there are no significant differences in the local heating effects. This result is in contrast with that obtained when comparing excitation at both wavelengths under CW excitation. We further demonstrate our observations by comparing the differences in a DNA tightrope denaturation experiment at 800 and 980 nm pulsed irradiation respectively, and find no significant change in denaturation at excitation fluences that have previously been shown to support upconversion imaging utilizing 976 nm excitation at the single particle level at diameters of 10 nm with an intensity of 3e4 W/cm^2^ and diameter of 25 nm at an intensity of 4e4 W/cm^2^ as demonstrated by Gargas et al. (2014) and Green et al. (2016).

## Experimental

### Synthesis of β-NaYF_4_: 20% Yb^3+^, 2% Er^3+^

The β-NaYF_4_:20%Yb, 2%Er UCNPs were prepared by combining 2.1 mmol of sodium trifluoroacetate, 0.78 mmol of yttrium trifluoroacetate, 0.2 mmol of ytterbium trifluoroacetate, and 0.02 mmol of erbium trifluoroacetate in 6 mL of oleic acid and 6.1 mL of octadecene. The solution was degassed at 120°C for 2 h with argon purging. Temperature was then increased to 330°C under argon and allowed to maintain this temperature for 25 min. The particles were then cooled, precipitated, washed in excess ethanol with centrifuging, and dried under vacuum.

### Synthesis of β-NaYF_4_:40%Yb, 2%Er@NaYF_4_:20%Yb@NaNdF_4_:10%Yb core-shell-shell UCNPs

The β-NaYF_4_:40%Yb, 2%Er Core UCNPs were prepared by a two-step thermolysis method. In the first step, CF_3_COONa (0.50 mmol), Y(CF_3_COO)_3_ (0.29 mmol), Yb(CF_3_COO)_3_ (0.20 mmol) and Er(CF_3_COO)_3_ (0.01 mmol) precursors were mixed with oleic acid (5 mmol), oleyamine (5 mmol), and 1-octadecene (10 mmol) in a two-neck round bottom flask. The mixture was heated to 110°C to form a transparent solution followed by 10 min of degassing. Then the mixture was heated to 300°C at a rate of 15°C/min under dry argon flow, and maintained at 300°C for 30 min to form the α-NaYF_4_:40%Yb, 2%Er intermediate UCNPs. After the mixture cooled to room temperature, the α-NaYF_4_:40%Yb, 2%Er intermediate UCNPs were collected by centrifugal washing with excessive ethanol (7,500 g, 30 min). In the second step, the α-NaYF_4_:40%Yb, 2%Er intermediate UCNPs were redispersed into oleic acid (10 mmol) and 1-octadecene (10 mmol) together with CF_3_COONa (0.5 mmol) in a new two-neck round bottom flask. After degassing at 110°C for 10 min, this flask was heated to 325°C at a rate of 15°C/min under dry argon flow, and maintained at 325°C for 30 min to complete the phase transfer from α to β. After the mixture cooled to room temperature, the β-NaYF_4_:40%Yb, 2%Er UCNPs were collected by precipitated with equal volume of ethanol and centrifugation afterwards (7,500 g, 30 min). The β-NaYF_4_:40%Yb, 2%Er UCNPs were stored in hexane (10 mL).

Next, the as-synthesized β-NaYF_4_:40%Yb, 2%Er core UCNPs served as cores for the epitaxial growth of core-shell UCNPs. A hexane stock solution of β-NaYF_4_:40%Yb, 2% Er core UCNPs was transferred into a two-neck round bottom flask, and the hexane was sequentially evaporated by heating. CF_3_COONa (0.50 mmol), Y(CF_3_COO)_3_ (0.40 mmol) and Yb(CF_3_COO)_3_ (0.10 mmol) were introduced as UCNP shell precursors with oleic acid (10 mmol) and 1-octadecene (10 mmol). After 10 min of degassing at 110°C, the flask was heated to 325°C at a rate of 15°C/min under dry argon flow and maintained at 325°C for 30 min to complete the shell crystal growth. After the mixture cooled to room temperature, the β-NaYF_4_:40%Yb, 2%Er@NaYF_4_:20%Yb core-shell UCNPs were collected by precipitated with equal volume of ethanol and centrifugation afterwards (7,500 g, 30 min). The β-NaYF_4_:40%Yb, 2%Er@NaYF_4_:20%Yb core-shell UCNPs were stored in hexane (10 mL).

Afterwards, the as-synthesized β-NaYF_4_:40%Yb, 2%Er@NaYF_4_:20%Yb core-shell UCNPs served as cores for the epitaxial growth of shell crystal. A hexane stock solution of β-NaYF_4_:40%Yb, 2%Er@NaYF_4_:20%Yb core-shell UCNPs was transferred into a two-neck round bottom flask, and the hexane was sequentially evaporated by heating. CF_3_COONa (0.50 mmol), Nd(CF_3_COO)_3_ (0.45 mmol) and Yb(CF_3_COO)_3_ (0.05 mmol) were introduced as UCNP shell precursors with oleic acid (10 mmol) and 1-octadecene (10 mmol). After 10 min of degassing at 110°C, the flask was heated to 325°C at a rate of 15°C/min under dry argon flow and maintained at 325°C for 30 min to complete the shell crystal growth. After the mixture cooled to room temperature, the β-NaYF_4_:40%Yb, 2%Er@NaYF_4_:20%Yb@NaNdF_4_:10%Yb core-shell-shell UCNPs were collected by precipitated with equal volume of ethanol and centrifugation afterwards (7,500 g, 30 min). The β-NaYF_4_:40%Yb, 2%Er@NaYF_4_:20%Yb@NaNdF_4_:10%Yb core-shell-shell UCNPs were stored in hexane (10 mL).

### Characterization and sample preparation

UCNP were characterized with Transmission Electron Microscopy (TEM) to determine average diameter. The β-NaYF_4_:20%Yb, 2%Er (Wirth et al., 2017; Figure 1b) and β-NaYF_4_:40%Yb, 2%Er@NaYF_4_:20%Yb@NaNdF_4_:10%Yb core-shell-shell (Figure 1c) UCNPs were imaged using a JEOL 2000FX TEM at the Analytical Instrumentation Facility on North Carolina State University's campus. Samples used for spectroscopic experimentation were created by diluting the concentration of UCNPs to 10 ng/mL in ethanol and pipetting 100 uL of solution onto the sample surface. The solution was shaken on the sample surface for 20 min before washing with ethanol and drying under nitrogen.

### Time-resolved spectroscopy measurements

Objects resolved on an optical microscope (40x, 0.9 N.A. air objective) imaged by a Andor NEO sCMOS camera with excitation from a 1,000 hz, tunable Nd: YAG laser with a 4.5 ns pulse width were selected by size, ensuring that imaged objects were less than the size of the diffraction limited spot of the wavelength of collection. Spectral distributions were separated using a half-meter monochromator with a custom exit with two slits. The gap between the slits correspond to a 10 nm wavelength difference centered at the monochromator's single slit location. By setting the monochromator to 535 nm, the 525 nm peak and 545 nm peak were effectively separated at the exit slits and further separated by a prism and coupled into separate SPCM-AQRH avalanche photodiodes. Controlled heating was performed using a peltier heater fixed to the back of the sample slide by epoxy. A thermistor attached to a PTC2.5K-CH temperature controller was embedded in the epoxy to ensure a constant set temperature during operation, and a K-type thermocouple was used to monitor the temperature at the sample surface.

### DNA tightrope measurements

The DNA substrates (CpG-free-rich, 7,163 bp) were ligated using the Quick Ligation Kit (New England BioLabs) at room temperature for overnight and then purified by phenol-chloroform extraction to remove the ligase (Pan et al., 2017). Coverslips were PEGylated prior to use Flow cells were assembled by using double-sided tape to attach PEGylated coverslips to microscope slides with drilled input holes. After assembling the flow cell, poly-L-lysine (Wako Chemicals) treated silica beads (D~5 um) were immobilized on the coverslips at a proper density and incubated for 5 min to ensure attachment. Afterwards, 100 uL of 5 ng/uL ligated DNA in a 7.4 pH buffer containing 50 mM HEPES, 100 mM NaCl, and 1 mM MgCl_2_ was injected into the flow cell and pushed back and forth for 10 min using a syringe pump at a flow rate of 300 uL/min (Pan et al., 2017). After the DNA was stretched between the beads, the DNA was stained with a YOYO-1 dye. Following that, the DNA tightropes were centered in the laser focal area and imaged for 10 s with a Xenon lamp at 70 mW/cm^2^. The DNA is then exposed to 976 nm at 7 × 10^4^ W/cm^2^ or 806 nm at 5 × 10^4^ W/cm^2^, for 2 min, followed by Xenon lamp imaging again for 10 s. A control DNA tightrope experiment without near infrared exposure was also performed alongside the near infrared exposed DNA tightropes.

## Result and discussion

**Figure 2:**
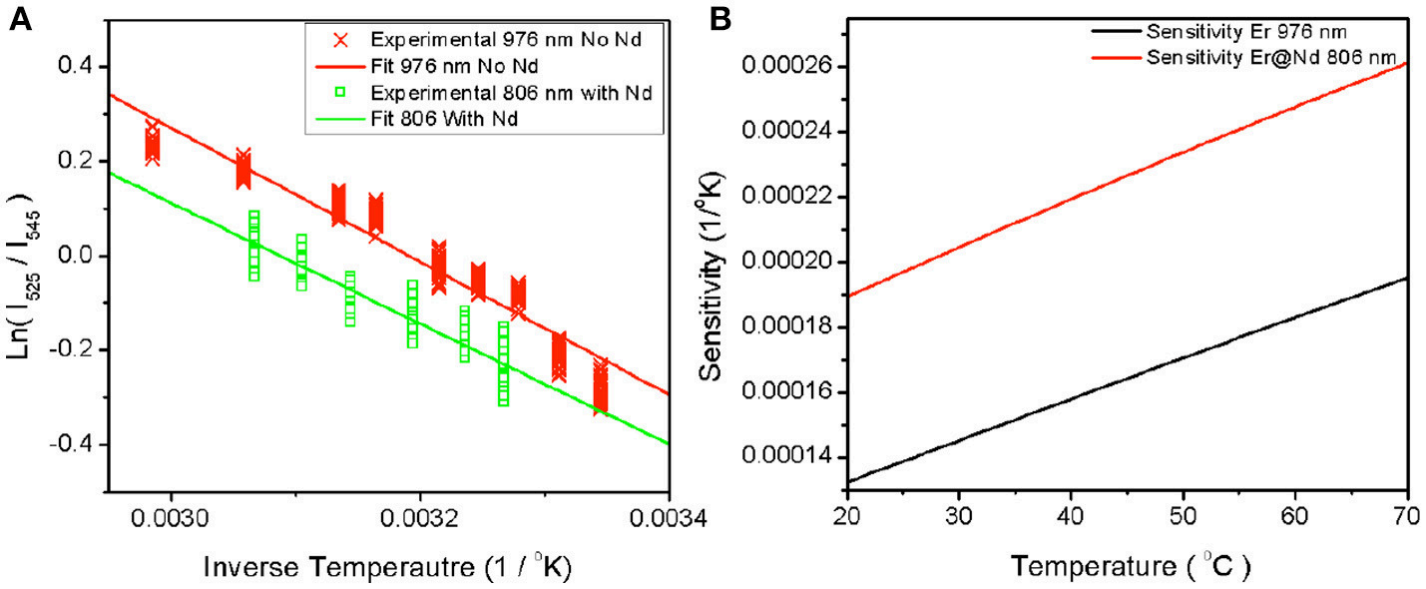
Plot of experimentally measured ratio against inverse temperature **(A)** for β-NaYF_4_:20%Yb, 2% Er excited at 976 nm and β-NaYF_4_:40%Yb, 2%Er@NaYF_4_:20%Yb@NaNdF_4_:10%Yb excited at 806 nm. The fit lines represent the slope between the natural log of the measured ratios and the inverse temperature. The sensitivity over the range of interest is plotted in **(B)** against temperature.

To establish the temperature sensing abilities of each type of UCNP under each excitation condition, controlled heating was used to bring the samples to set temperatures as described in the methods section. Spectroscopic measurements were performed at each temperature after a period of equilibration of 10 min. Figure 2 shows a plot of ln(I_525_/I_545_) vs. 1/T measured for the both the β-NaYF_4_:20%Yb, 2%Er UCNPs and β-NaYF_4_:40%Yb, 2%Er@NaYF_4_:20%Yb@NaNdF_4_:10%Yb core-shell-shell UCNPs at 976 and 806 nm respectively. Since the population of the Er: ^2^H_11/2_ and Er: ^4^S_3/2_ levels fluctuates as a function of the Boltzmann's distribution (Lei et al., 2005),

$$\text{R}=\frac{{\text{I}}_{525}}{{\text{I}}_{545}}=\text{Λ}{\text{e}}^{\frac{-\text{ΔE}}{\text{kT}}}$$

where by taking the slope of the ln(I_525_/I_545_) vs. 1/T plot, a ΔE of 887.170 cm^−1^ (806 nm, core-shell-shell with Nd) and 966.176 cm^−1^ (976 nm core only) is obtained. The calculated difference between 545 and 525 nm peaks is 700 cm^−1^. The sensitivity S is defined as,

$$\text{S}=\frac{\text{d}(\text{R})}{\text{dT}}\text{Λ}\frac{\text{ΔE}}{\text{k}{\text{T}}^{2}}{\text{e}}^{\frac{-\text{ΔE}}{\text{kT}}}$$

where the higher the temperature, the greater the sensitivity. Given the calculated ΔE, a plot of the sensitivity against temperature is shown in Figure 2B. Our sensitivity values are comparable to other researchers (León-Luis et al., 2012; Table 1). The higher sensitivity expands the applicability to environmental and electronics sensing where typical critical operating temperatures are higher.

**Table 1:** The natural log of the ratio between emissive states was plotted against inverse temperature as seen in Figure 2.

**Parameter**	**No Nd 976**	**No Nd 800**	**With Nd 976**	**With Nd 800**
Delta E (cm^−1^)	966	1,492	1,288	887
Slope	−1,413	−2,147	−1,768	−913
Intercept	4.51	6.485	4.984	2.971
Sensitivity at 25°C	1.4*E*−04	1.8*E*−05	5.28*E*−05	4.8*E*−04
Sensitivity at 60°C	1.8*E*−04	3.07*E*−05	7.88*E*−05	5.3*E*−04

*This table is the result of fitting the resulting curve. The slope is directly equivalent to Delta E over k*.

Local temperature rise with continuous laser irradiation at high pump power intensity and duration has been observed (Wang et al., 2013), which can potentially impact *in vivo* applications. To address these concerns, we investigated the influence of the irradiation pump intensity and duration. To investigate the feasibility of experiments utilizing high laser fluence combined with pulsed excitation, we investigate the change in the spectroscopic ratio, with laser intensity and time duration in air and water.

**Figure 3:**
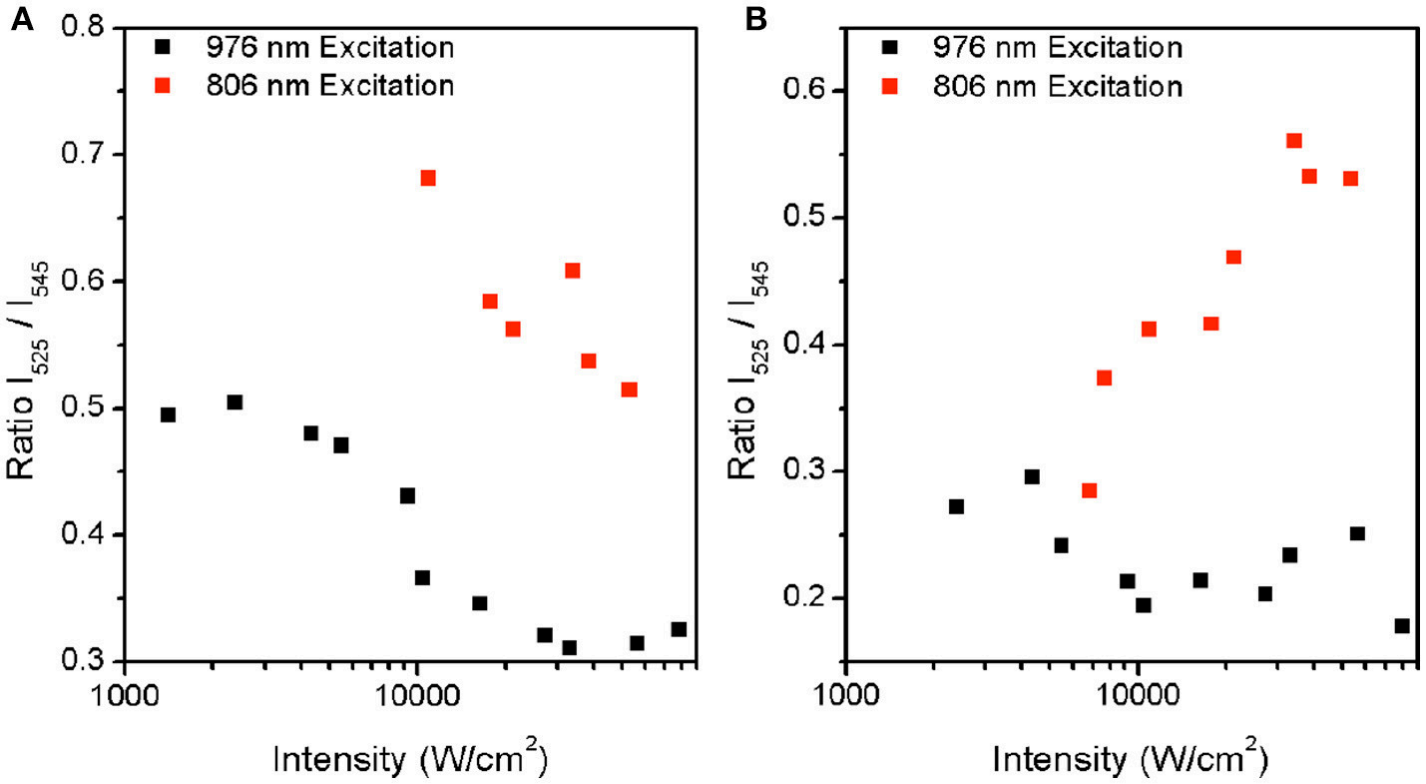
Plot of measured ratio between ^2^H_11/2_ and ^4^S_3/2_ at increasing intensity for both 806 nm and 976 nm excitation for **(A)** β-NaYF_4_:20%Yb, 2% Er **(A)** and **(B)** β-NaYF_4_:40%Yb, 2%Er@NaYF_4_:20%Yb@NaNdF_4_:10%Yb.

**Figure 4:**
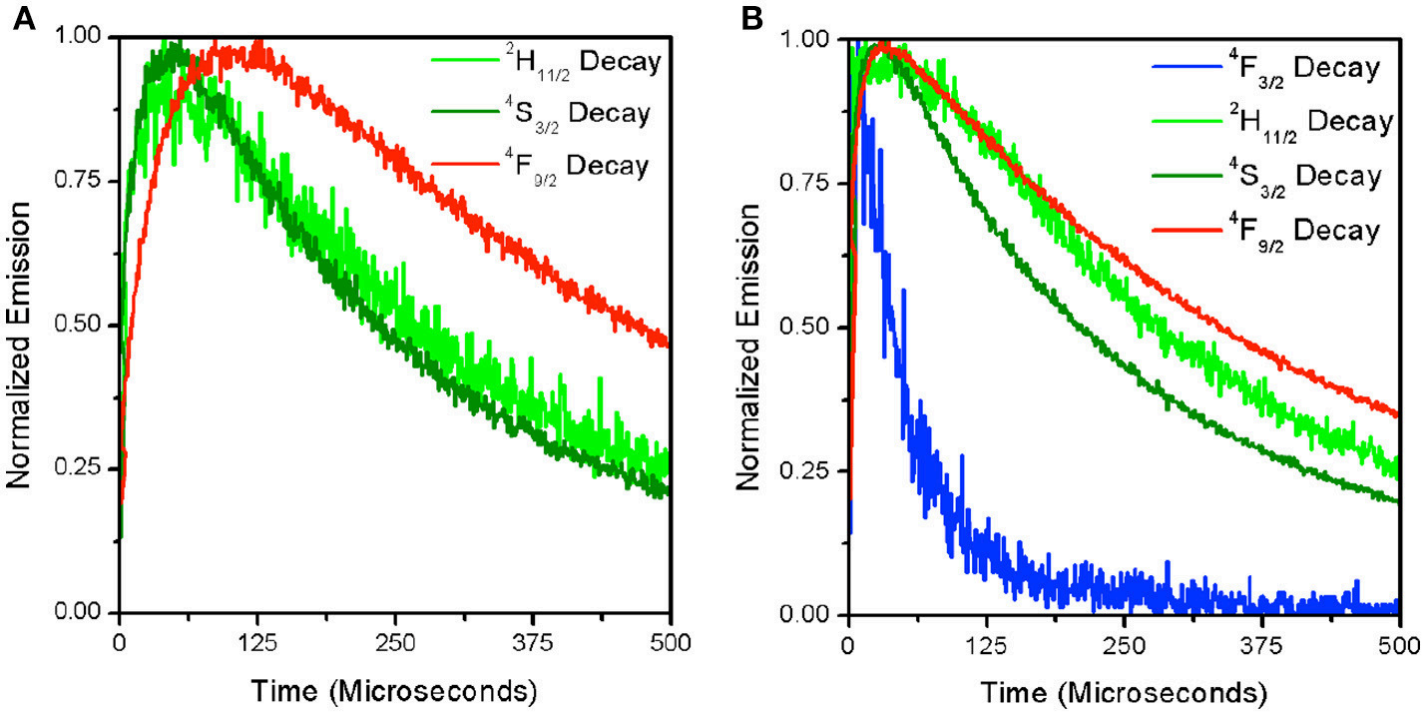
Time-resolved decay for each of the visible emitting levels for β-NaYF_4_:20%Yb, 2% Er at 1.0 × 10^4^ W/cm^2^ **(A)** and 5.6 × 10^4^ W/cm^2^ **(B)**. The ^4^F_3/2_ decay is only shown in the right panel where signal intensity was sufficient to produce a readable curve.

Figure 3 shows the dependence of the spectroscopic ratio, R, on the pump power intensity. There is a marked difference in the optical response of the particles with respect to near infrared excitation wavelength. In the β-NaYF_4_:20%Yb, 2%Er UCNPs (Figure 3A), R is observed to decrease with increasing pump power intensity at 976 nm excitation. This decrease in ratio apparently contradicts an expected rise in R in the presence of potential local heating. This is expected as a higher pump power intensity enables transition from 2 photon to 3 photon upconversion, where increasing pump power intensity leads to population of the ^4^G_11/2_, after which phonon relaxation to the ^2^H_9/2_ level occurs. Subsequently radiative relaxation from the ^2^H_9/2_ to ^4^I_15/2_ level results in blue emission, as shown in Figure 4B. Thereby, due to preferential population of the ^4^G_11/2_ level, we expect to observe a lower photon population of the ^2^H_11/2_ (525 nm) level, at high pump power intensities. As the pump power is increased, the increase in phonon coupling to the lattice, and subsequent non-radiative energy transfer from the ^2^H_9/2_ to ^2^H_11/2_ and the ^4^S_3/2_ level to the ^4^F_9/2_ level occurs. Since the energy gap for the ^4^S_3/2_ to ^4^F_9/2_ transition of 3117 cm^−1^ coincides with the typical value of 3,000–3,600 cm^−1^ for OH vibrations(Kim et al., 2017), a higher pump power results in greater non-radiative relaxation via this pathway, as seen by the decrease in the rise and decay time of the Er^3+^: ^4^F_9/2_ to ^4^I_15/2_ transition (Figures 2–4 and Table 2). In comparison, the energy gap for the Er^3+^: ^2^H_9/2_ to ^2^H_11/2_ transition of around 6000 cm^−1^, is much larger than that of the OH absorption energy. The resulting effect of a higher pump power is to promote greater blue and red emissions at the expense of green emission. A similar, but less dramatic, decrease in R is also seen at 806 nm excitation, where the absorption of two photons populates the ^2^H_9/2_ and subsequent radiative relaxation to the ^4^I_15/2_ level results in blue emission. We note that the absorption cross-section at 806 nm is comparatively low for this sample. However, increased pump power intensity at 806 nm did further increase blue emission, while also increasing rates of non-radiative transfer, leading to population of the ^4^F_9/2_ state. Therefore, in the β-NaYF_4_:20%Yb, 2%Er UCNPs, if used as a temperature sensor at either 806 nm or 976 nm excitation, blue emission is triggered at pump power intensities, which affects the ^2^H_11/2_ (525 nm) level population significantly and the ratio decrease should be accounted for.

**Table 2:** Lifetime and rise times fitted from the time-resolved decay for β-NaYF_4_:20% Yb^3+^, 2% Er^3+^ from Figure 4.

	**Blue**	**H**	**S**	**Red**
5.6 × 10^4^ W/cm^2^				
Lifetime	53.792	352.113	267.308	432.339
Rise Time	13.125	20.125	35.000	35.000
1.0 × 10^4^ W/cm^2^				
Lifetime	N/A	344.471	272.405	558.971
Risetime	N/A	45.500	59.500	91.875

*The lifetime was fitted as a single exponential for the emission's decay and the rise time was defined as the time between the laser excitation and the maximum point of the decay curve*.

In Figure 3B, the β-NaYF_4_:40%Yb, 2%Er@NaYF_4_:20%Yb@NaNdF_4_:10%Yb core-shell-shell UCNPs, at 976 nm excitation, shows a drop in R, but to a smaller degree, with increasing pump power intensity. This is attributed similarly to the β-NaYF_4_:20%Yb, 2%Er UCNPs as shown earlier. The smaller drop may be due to the protective nature of the core-shell-shell configuration, which limits the effect of lattice vibration effects. In contrast, at 806 nm excitation, an increase in R is seen with increasing pump power. The immediate assumption is that of an increase in local temperature, but results shown later will demonstrate that this is not the case. The increase in R appears to be an optical effect only. Under 806 nm excitation, the Nd^3+^: ^4^F_5/2_ and Nd^3+^: ^2^H_9/2_ levels are populated due to transitions from the Nd^3+^:^4^I_9/2_ ground state. Subsequent non-radiative relaxation from these two states occur, leading to population of the Nd^3+^: ^4^F_3/2_ state. The resonant energy levels of Nd^3+^: ^4^F_3/2_ and Yb^3+^: ^2^F_5/2_ ensures a very high efficiency energy transfer (ET) between Nd^3+^ ions and Yb^3+^ ions(Wang et al., 2013; Tian et al., 2014; Zhong et al., 2014; Chen et al., 2015). Thereafter, two consecutive energy transfers from the Yb^3+^: ^2^F_5/2_ state to the neighboring Er^3+^ ions, result in population of the ^4^F_7/2_ state. Relaxation from the ^4^F_7/2_ state to the lower ^2^H_11/2_, ^4^S_3/2_ and ^4^F_9/2_ states, followed by radiation relaxation from all three of states to the ^4^I_15/2_ ground state, give rise to emission at 525 nm, 545 nm, and 655 nm, respectively. Since the highly absorbing Nd^3+^ ion promotes more efficient energy transfer to the Yb^3+^ ion, a higher pump power intensity also leads to a more efficient population of the ^4^F_7/2_ level, where further non-radiative relaxation to the ^2^H_11/2_ state occurs, giving rise to preferential emission at 520 nm. Therefore, the Nd^3+^ dopant, due to the larger absorption cross-section, and more efficient energy transfer to the Yb^3+^ ion, clearly favors upconversion over linear decay. As a result, an increase in laser intensity, leads to greater upconversion.

To further understand the source of the decrease in R with increasing excitation intensity for the β-NaYF_4_:20%Yb, 2%Er UCNPs, time-resolved decay spectra were measured. Figures 4A,B displays the decay spectra for ^4^F_3/2_(blue), ^2^H_11/2_(green), ^4^S_3/2_(green), and ^4^F_9/2_(red) emissions at two different excitation intensities. It can be seen that at a higher excitation intensity (5.6 × 10^4^ W/cm^2^) in Figure 4B, that the ^4^F_9/2_ and ^4^S_3/2_ states excitation pathways are more strongly coupled as evidenced by the narrowing of the gap between their rise times (See Table 2). This has been attributed to the increase in phonon coupling to the lattice OH vibrations at higher laser intensity. At lower laser intensity, the ^4^F_9/2_ rise time is significantly longer in comparison to that of the ^4^S_3/2_ state. Supporting Figure S1 shows normalized time-resolved decay at 545 nm of the β-NaYF_4_:20%Yb, 2%Er UCNPs and β-NaYF_4_:40%Yb, 2%Er@NaYF_4_:20%Yb@NaNdF_4_:10%Yb core-shell-shell UCNPs. Supporting Figures S1A,B show the 545 nm decay of the Yb/Er co-doped sample from excitation at 976 nm and 806 nm respectively. In Supporting Figure S1A, the slow rise times and long decay times are indicative of the ETU upconversion process. As the pump power intensity is increased, the rise times and mean lifetimes are both reduced. The corresponding emission at 806 nm excitation is dim, due to the low absorption cross-section at this excitation wavelength, hence the decay curves are noisy, and convey less information. Supporting Figures S1C,D shows the decay curves at 545 nm for the core-shell-shell UCNPs for excitation at 976 nm and 806 nm respectively. Both figures clearly show faster rise times and decay, which we attribute, for the 976 nm excitation, to the presence of core-shell-shell nanostructure, which reduces phonon coupling to the host matrix. At 800 nm excitation, the highly absorbing Nd^3+^ ion further increases the decay rate for the Er^3+^: ^4^S_3/2_ to ^4^I_15/2_ transition.

**Figure 5:**
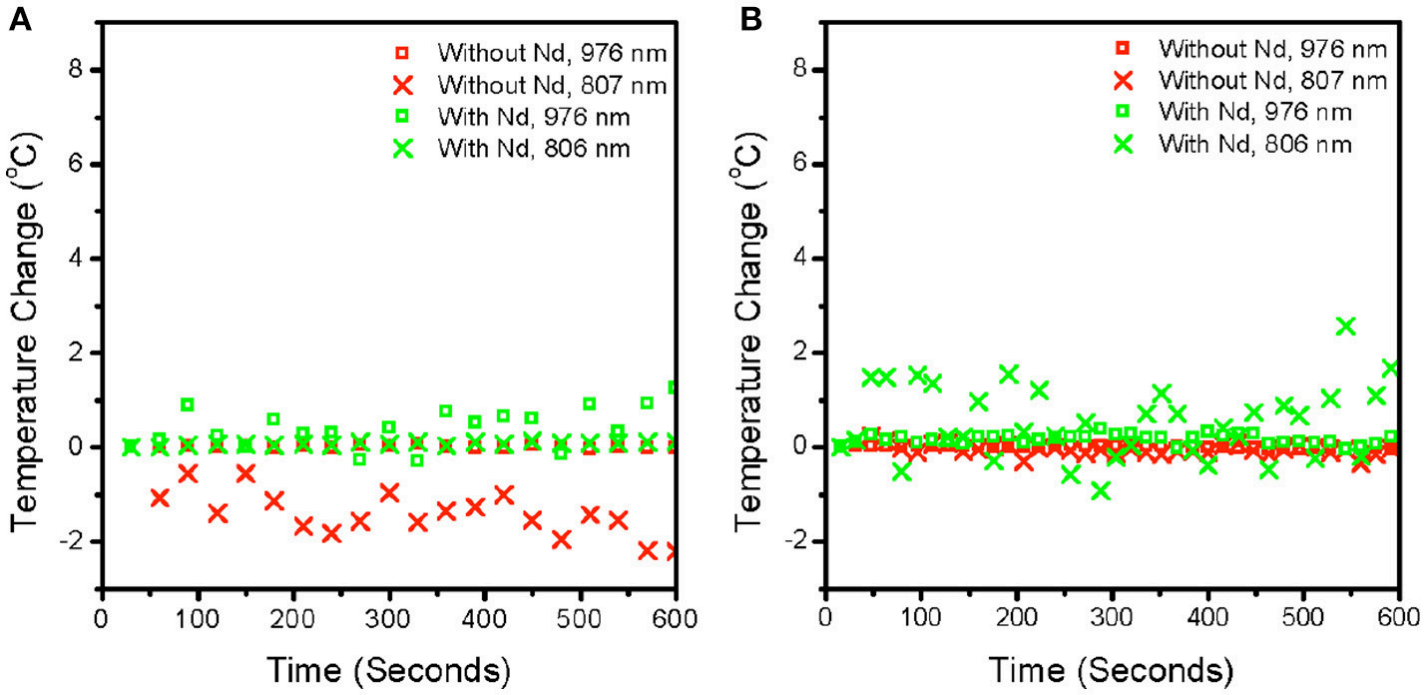
Plot of measured temperature converted from spectroscopic ratio against time at a fixed power for particles without Nd (β-NaYF_4_: 20% Yb^3+^, 2% Er^3+^) and with Nd (β-NaYF_4_: 40%Yb^3+^, 2% Er^3+^@NaYF_4_: 20% Yb^3+^@NaNdF4: 10% Yb^3+^) in air **(A)** and in water **(B)**. The laser was blocked for 10 min before the beginning of the measurement and unblocked at around 20 s to collect any changes in temperature after the sample was exposed. The powers used were 8.0 × 10^4^ W/cm^2^ and 5.3 × 10^4^ W/cm^2^ for 976 and 806 nm respectively. Both power intensities were maintained at comparable orders of magnitude as much as possible. The pulse width is ~4.5 ns and pulse frequency is at 1,000 hz. Each point is at 16 s intervals, so each point is the sum of 16 k pulses.

Since it is unclear as to the impact of pump power intensity on local heating due to the increased probability of higher energy level transitions, we investigated the effect of time duration of excitation on the local temperature rise. Figures 5A,B show the dependence of the local temperature with time duration of irradiation in air (Figure 5A) and water (Figure 5B), respectively. Neither 976 nm, nor 806 nm excitation introduces a local temperature rise over time at laser intensities in the range of 10^4^ W/cm^2^. Our results are significantly different from other researchers primarily because our experiments are conducted with pulsed excitation, unlike the effects seen using continuous irradiation as observed by other researchers (Wang et al., 2013). Therefore, the laser mode of operation should be carefully considered regarding the use of upconversion nanocrystals in thermal sensing. While continuous wave lasers are affordable and highly adaptable to many laboratories, the probability of local heating is high. In contrast, pulsed wave excitation under a tightly focused beam, which is typical in single-molecule imaging does not cause appreciable local temperature rise in the sample.

**Figure 6:**
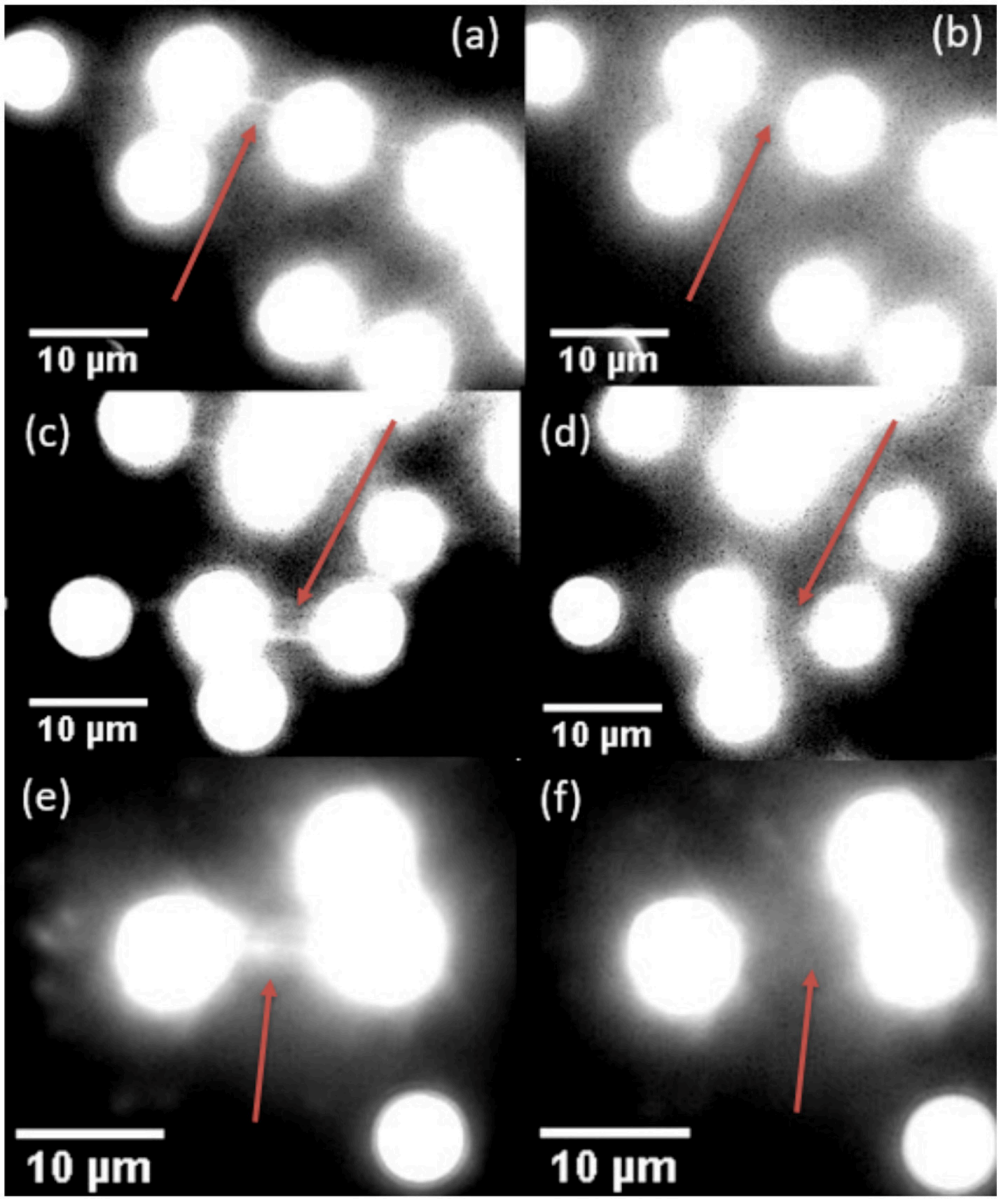
Examples of DNA tightropes before and after breaking under 806 nm fluence **(a,b)**, 976 nm fluence **(c,d)**, and with no applied fluence **(e,f)**. In all three cases, the left image was taken upon the initial location of the DNA tightrope. The lamp was then blocked, and the right image was taken after exposure to 806 nm **(b)**, 976 nm **(d)**, or no laser fluence **(f)** for 2 min.

As a demonstration of the effects of 976 vs. 806 nm irradiation at the single-molecule level, we compared DNA tightropes exposed to both wavelengths at intensities shown previously to support single particle imaging (Gargas et al., 2014; Green et al., 2016). In order to clearly identify effects of near infrared exposure, we first stretch DNA on silica beads by flowing the DNA across the beads in an aqueous environment. After the DNA are stretched out and stained with YOYO-1 dye, the laser spot is localized on the DNA tightrope. The tightropes are then exposed to 806 nm or 976 nm excitation separately at power intensities compatible with single UCNP imaging as demonstrated in previous studies (Gargas et al., 2014; Green et al., 2016). Figure 6 shows that exposure to 800 nm (5 × 10^4^ W/cm^2)^ and 976 nm (7 × 10^4^ W/cm^2^) excitation for 2-min durations only cause dsDNA breaks 10% of the time. For a control DNA tightrope experiment where there was no exposure to near infrared excitation, the rate of occurrence of dsDNA breaks were also around 10%. Therefore, any damage observed is consistent with 10 s of UV or blue light nicking of the DNA, from the Xenon Arc Lamp source used to detect YOYO-1 emission. Clearly, from our measurements, the lack of DNA damage under infrared exposure leads us to infer that thermal heating is not significant at the current pump power intensities used in our measurements especially when considered alongside the lack of heating in water, as shown in Figure 5.

## Conclusion

UCNPs with and without Nd^3+^ sensitization may be good candidates for application as nanothermometers in single biological molecule experimental situations. It was demonstrated that each type of UCNP was able to report temperature ratiometrically with sensitivities in the 1 × 10^−4^ K^−1^ range. In addition, it was determined that excitation intensity is a parameter of nanothermometry that must be strongly controlled in the single particle level to avoid ratio suppression due to alteration of the optical pathways involved in ratio reporting. Finally, the excitation intensities previously used to image single nanoparticles were shown to be applicable to DNA tightrope experiments without appreciable change in the viability of DNA over time windows appropriate for the study of protein-DNA interactions.

## Author contributions

The study aims were proposed by SL. KH synthesized the UCNPs. KG designed and performed the spectroscopic experiments. HP prepared the biologicals for the DNA tightrope experiments. Data interpretation and analysis were performed by KG, KH, GH, and SL.

### Conflict of interest statement

The authors declare that the research was conducted in the absence of any commercial or financial relationships that could be construed as a potential conflict of interest.

## Supplementary material

Supplementary Video 1 Intensity varies as nanoparticles tumble in the laser spot due to polarization sensitivity. Seen here, single nanoplasmonic UCNPs tumble in 50% sucrose solution, recorded at a frame rate of 10Hz.

Supporting Figure S1 Time-resolved decay curves at 545 nm for β-NaYF_4_:20%Yb, 2%Er at **(A)** 976 nm, and **(B)** 807 nm excitation respectively, and β-NaYF_4_:40%Yb, 2%Er@NaYF_4_:20%Yb@NaNdF_4_:10%Yb at **(C)** 976 nm, and **(D)** 806 nm excitation respectively. In frame **(D)** the sample was partially removed from the collection area, resulting in lower signal, noisy collection.
